# On the limits of observing motion in time-resolved X-ray scattering

**DOI:** 10.1098/rsta.2017.0477

**Published:** 2019-04-01

**Authors:** Matthew R. Ware, James M. Glownia, Adi Natan, James P. Cryan, Philip H. Bucksbaum

**Affiliations:** 1 National Accelerator Laboratory, Stanford PULSE Institute, Menlo Park, CA 94025, USA; 2 Department of Physics, Stanford University, Stanford, CA 94305, USA; 3 Department of Applied Physics, Stanford University, Stanford, CA 94305, USA; 4 National Accelerator Laboratory, LCLS, SLAC, Menlo Park, CA 94025, USA

**Keywords:** time-resolved X-ray scattering, molecular movies, X-ray free electron lasers

## Abstract

Limits on the ability of time-resolved X-ray scattering (TRXS) to observe harmonic motion of amplitude, *A* and frequency, *ω*_0_, about an equilibrium position, *R*_0_, are considered. Experimental results from a TRXS experiment at the LINAC Coherent Light Source are compared to classical and quantum theories that demonstrate a fundamental limitation on the ability to observe the amplitude of motion. These comparisons demonstrate dual limits on the spatial resolution through *Q*_max_ and the temporal resolution through *ω*_max_ for observing the amplitude of motion. In the limit where *ω*_max_ ≈ *ω*_0_, the smallest observable amplitude of motion is *A* = 2*π*/*Q*_max_. In the limit where *ω*_max_≥2*ω*_0_, *A*≤2*π*/*Q*_max_ is observable provided there are sufficient statistics.

This article is part of the theme issue ‘Measurement of ultrafast electronic and structural dynamics with X-rays’.

## 1. Introduction

Time-resolved X-ray and electron scattering (TRXS and TRES) have the potential to track nuclear and even electronic motion in molecules with atomic-level spatial and temporal resolution [1–3]. In TRXS experiments, the common current practice is to acquire nuclear positions by fitting scattering data to a theoretical model [4–6]. This reliance on theory presents a challenge when working to interpret published experimental data. For example, a model fit might resolve periodic motion with an amplitude of 0.005 Å in an experiment with 1.8 Å of diffraction limited resolution [6]. When is such a result statistically valid? Nuclear positions can also be obtained via de novo methods, i.e. by directly inverting the time-dependent X-ray scattering measured in momentum space into real space [2,3,7–9]. These methods raise additional questions. For example, the signal-to-noise ratio of an experiment can limit the fidelity of reconstructed nuclear positions, and incomplete information in the X-ray scattering pattern can lead to artefacts in the reconstructed real-space movies of nuclear motion. When interpreting TRXS results using these two approaches, we require a rule analogous to the diffraction limit for static imaging (*d*≥2*π*/*Q*_max_) to determine if a result is reasonable or significant. Some prior work has been done to place limits on the ability of TRXS to resolve motion. For example, Budarz *et al.* [4] discuss the blurring of the scattering pattern due to the finite scattering length of the sample cell, and Kirrander & Weber [10] discuss the limited resolution that arises due to the nature of the excited molecular wavepacket as opposed to the limitations of TRXS itself. Here we investigate the physical limits imposed by the measurement method itself. The goal is to establish criteria for the resolving power of TRXS to measure periodic motion with amplitude, *A*, about an equilibrium position, *R*_0_.

## 2. Experiment and theory

**Figure 1.**
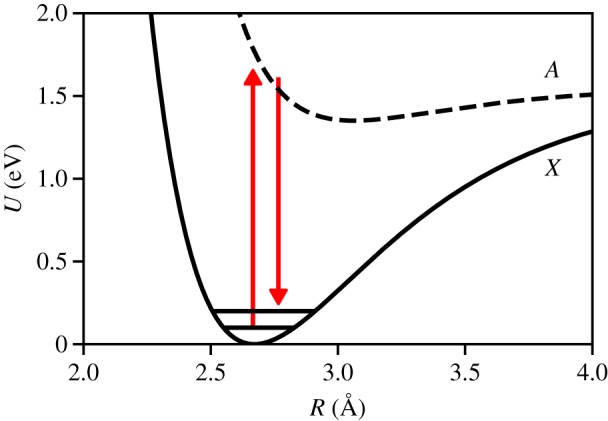
A strong 800 nm pump drives the Raman transition from and back onto the ground *X* state of iodine creating a vibrational wavepacket in the harmonic region of the *X* state. (Online version in colour.)

**Figure 2.**
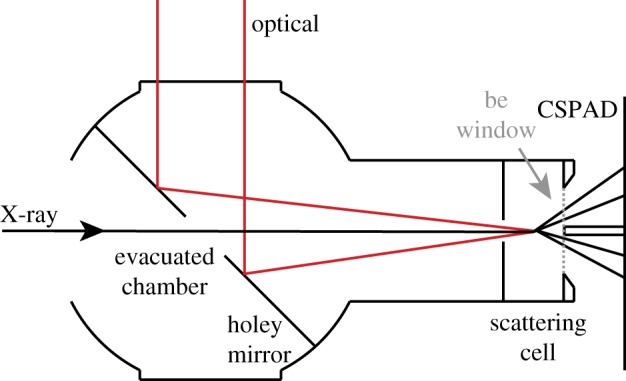
Within an evacuated chamber the incident X-ray pulse passes through a holey mirror. The optical pump reflects from the holey mirror and copropagates to the scattering cell alongside the X-ray pulse. The optical and X-ray pulses are focused onto the *I*_2_ sample within the scattering cell. The X-rays scatter from the sample with an intensity profile which is sensitive to the delay between the optical pump pulse and the incident X-ray pulse. The scattered X-rays then travel freely to the Cornell Stanford Pixel Area Detector (CSPAD) through the output berrilium window. (Online version in colour.)

**Figure 3.**
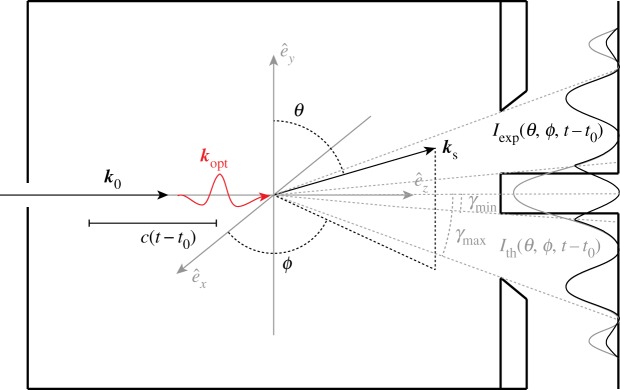
Sketch of the scattering cell and the scattering geometry of the experiment. *γ*_min_ and *γ*_max_ are the minimum and maximum scattering angles collected at the detector. Beyond *γ*_min_ and *γ*_max_, the measured intensity is attenuated relative to the predicted intensity profile. (*θ*, *ϕ*) is the relevant angular decomposition about the laser polarization axis, ${\hat{e}}_{y}$, as the molecules align along this axis. ***k***_0_ and ***k***_opt_ are the incident X-ray and optical wavevectors, respectively. Because the X-rays may scatter anywhere within the cell, the obtained scattering image is blurred by Δ*γ* = 3.4° (i.e. 0.26 Å^−1^ in *Q*-space), see [4]. (Online version in colour.)

**Figure 4.**
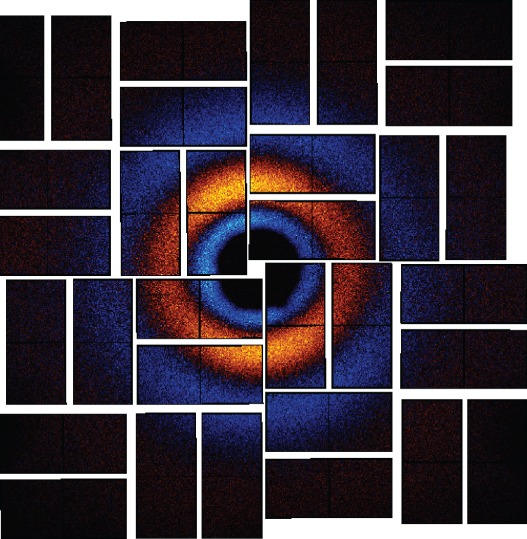
Difference image at the X-ray detector (CSPAD) for a delay of approximately 100 fs after pumping with 800 nm light.

The experiments under consideration are scattering from homonuclear diatomic molecules in the gas phase. Photoexcitation of molecular iodine, *I*_2_, is a particular focus for which we have collected data to compare with our findings. Perhaps the simplest motion that we can study is the coherent oscillation of molecular iodine in its ground electronic state following Raman excitation with 800 nm light as shown in figure 1. The ground state of *I*_2_ has been well-characterized by spectroscopy to have an equilibrium separation of 2.666 Å and a harmonic period of 155.5 fs [11–13], and pump-probe spectroscopies have directly observed vibrational wavepackets on the ground state [14]. We have studied the ground state motion of molecular iodine in the gas phase using the TRXS apparatus depicted in figure 2. The 800 nm pump pulse induces coherent motion (Δ*v* ± 1 [15]) of the individual iodine molecules, then the X-ray probe pulse arrives at variable delay, *τ* = *t* − *t*_0_, and scatters onto the two-dimensional (*x*, *y*) detector as shown schematically in figure 3 and discussed in detail in appendix A. In any TRXS experiment, the direct observable is the delay-dependent scattered X-ray intensity, *I*(*x*, *y*, *τ*), on the two-dimensional detector as shown in figure 4. The molecular scattering factor, *S*(*x*, *y*, *τ*), is extracted from *I*(*x*, *y*, *τ*) as described in appendix B. *S*(*x*, *y*, *τ*) is the key observable for determining interatomic distances in a TRXS experiment. To obtain interatomic distances, the pixel coordinates (*x*, *y*) at the detector must be mapped onto the momentum transfer, ***Q*** = ***k***_0_ − ***k***_*s*_, which is done using the scattering angle, *γ*, shown in figure 3, to find *Q* = 2*k*_0_sin(*γ*/2). Here the molecular scattering factor is angularly integrated at *Q* to obtain the isotropic scattering factor, *S*(*Q*, *τ*).

The isotropic molecular scattering factor is related to the nuclear probability density, *ρ*(*R*, *τ*), through a spatial transform, i.e.

2.1 $$S(Q,\tau )=\int dRR^{2}\;\mathrm{sinc}(QR)\rho (R,\tau ),$$

where *R* is the internuclear separation and *τ* is the delay between the optical-pump and X-ray-probe pulses.

**Figure 5.**
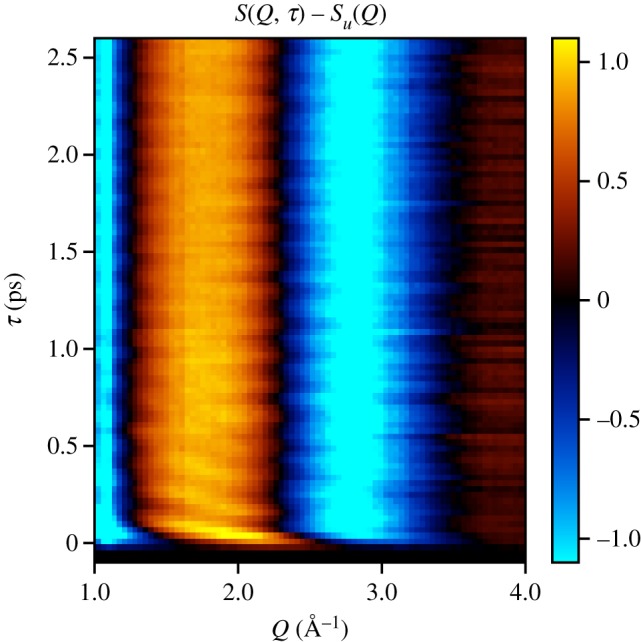
Time-resolved X-ray scattering following photoexcitation by an 800 nm pump pulse. This is the isotropic component of the molecular scattering factor. A fast 155.9 ± 3.9 fs beat is observed, corresponding to the harmonic period of the ground state of iodine. The unpumped scattering signal, *S*_*u*_(*Q*), is subtracted from this image.

The measured *S*(*Q*, *τ*) following the 800 nm pump pulse is shown in figure 5. Following some fast dissociation near *t*_0_, the dominant signal is a fast beat with a period of 155.9 ± 3.9 fs. The beat period is consistent with the previously measured harmonic period of the ground state of iodine, 155.5 fs, from [11,12].

While *S*(*Q*, *τ*) is the starting point for de novo methods and may be directly inverted like

2.2 $$\rho (R,\tau )=\int dQQ^{2}\;\mathrm{sinc}(QR)S(Q,\tau )$$

as shown in [9], the clear oscillations on the *X* state motivate an analysis of the temporal TRXS spectrum. Simple harmonic motion like *R*(*τ*) = *R*_0_ + *A*cos(*ω*_0_*τ* + *ϕ*) may be leveraged to invert the scattering pattern by first taking a temporal Fourier transform of the measured scattering pattern, i.e.

2.3 $$\tilde{S}(Q,\omega )=\int_{-\infty }^{+\infty }d\tau \;e^{-i\omega \tau }S(Q,\tau ).$$

**Figure 6.**
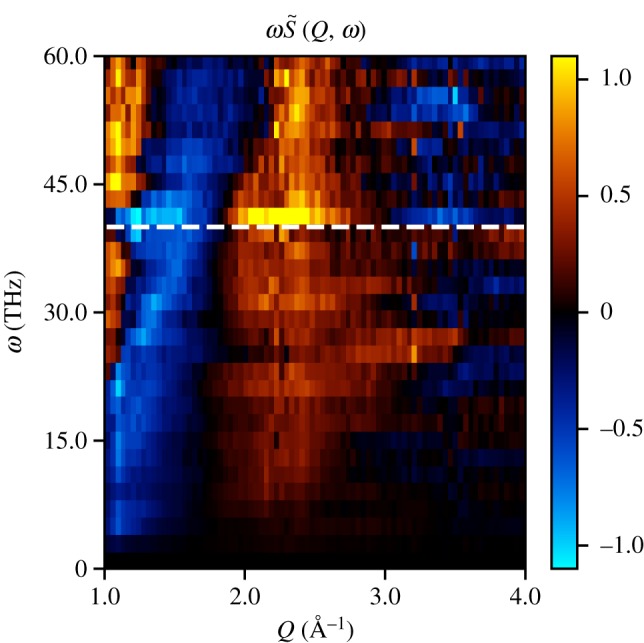
Temporal Fourier transform of figure 5. We observe a strong frequency component at 40.3 ± 1.0 THz corresponding to the harmonic frequency of the ground state of iodine. The lineout at this frequency may be used to obtain the equilibrium separation of the atoms in motion as shown in figure 7.

From the measured TRXS, *S*(*Q*, *τ*), the harmonic motion of iodine in its ground state may be isolated by taking a temporal Fourier transform, $\tilde{S}(Q,\omega )$, as shown in figure 6. To gain insight into the TRXS spectrum at the harmonic frequency of 40.3 ± 1.0 THz, a classical model for $\tilde{S}(Q,\omega )$ using simple harmonic motion is now derived.

**Figure 7.**
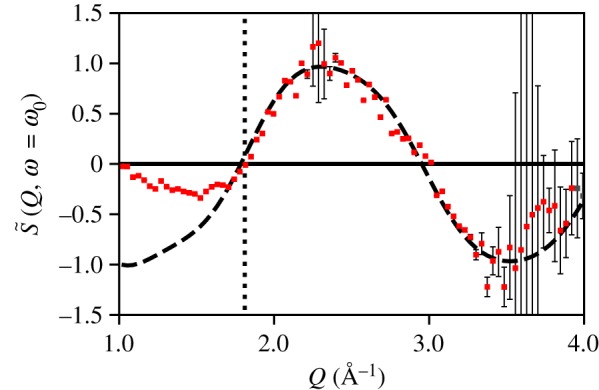
Lineout of figure 6 as compared to the classical theory for scattering from a vibrating diatomic. The error bars arise from a shot-by-shot statistical analysis and do not account for systematic effects. The statistical error increases at high *Q* where the measured intensity is low. Below the dashed line at 1.81 Å^−1^, the signal is attenuated due to a physical beam block as shown in figure 3. (Online version in colour.)

Classical isotropic time-resolved X-ray scattering may be expressed as

2.4 S(Q,t)=sinc QR(t),

from [16]. The above equation may be rewritten as

2.5 $$\begin{array}{rl} S(Q,t) & =\frac{1}{Q}\int_{0}^{Q}dQ^{\prime }\cos\left(Q^{\prime }R(t)\right)\\ & =\frac{1}{2Q}\int_{0}^{Q}dQ^{\prime }\left[e^{iQ^{\prime }R(t)}+e^{-iQ^{\prime }R(t)}\right]\\ & =\frac{1}{2Q}\left(I_{+}+I_{-}\right). \end{array}$$

Then,

2.6 $$\tilde{S}(Q,\omega )=\frac{1}{2Q}\left({\tilde{I}}_{+}+{\tilde{I}}_{-}\right),$$

where

2.7 $${\tilde{I}}_{\pm }=\frac{1}{2\pi }\int_{0}^{Q}dQ^{\prime }\int_{-\infty }^{+\infty }dt\;e^{\pm iQ^{\prime }R(t)-i\omega t}.$$

Laser initiated periodic motion may be expressed as a piece-wise function, where *R*(*t*) = *R*_0_ for *t*≤*t*_0_ and *R*(*t*) = *R*_0_ + *A*cos(*ω*_0_*t* + *ϕ*) for *t* > *t*_0_. To obtain an analytic solution, the time-range of motion must be extended from (*t*_0_, ∞) to ( − ∞, ∞). Using *R*(*t*) = *R*_0_ + *A*cos(*ω*_0_*t* + *ϕ*) for *t*∈( − ∞, ∞),

2.8 $${\tilde{I}}_{\pm }=\frac{1}{2\pi }\int_{0}^{Q}dQ^{\prime }\;e^{\pm iQ^{\prime }R_{0}}\int_{-\infty }^{+\infty }dt\;e^{\pm iQ^{\prime }A\cos(\omega_{0}t+\phi )-i\omega t}.$$

In the limit *QA*≪1, the exponential expands to first order as

2.9 $$e^{\pm iQ^{\prime }A\cos(\omega_{0}t+\phi )}\approx 1\pm iQ^{\prime }A\cos(\omega_{0}t+\phi ).$$

Ignoring the addition of 1 as it only contributes to DC, equation (2.9) evaluates as follows:

2.10 $$\begin{array}{rl} {\tilde{I}}_{\pm } & \approx \frac{\pm iA}{4\pi }\int_{0}^{Q}dQ^{\prime }Q^{\prime }\;e^{\pm iQ^{\prime }R_{0}}\int_{-\infty }^{+\infty }dt\left(e^{i\omega_{0}t+i\phi -i\omega t}+e^{-i\omega_{0}t-i\phi -i\omega t}\right)\\ & \approx \frac{\pm iA}{2}\int_{0}^{Q}dQ^{\prime }Q^{\prime }\;e^{\pm iQ^{\prime }R_{0}}\left(e^{i\phi }\delta (\omega -\omega_{0})+e^{-i\phi }\delta (\omega +\omega_{0})\right)\\ & \approx \frac{\pm iA}{2}\left[-\frac{1}{R^{2}}+e^{\pm iQR_{0}}\left(\frac{1}{R_{0}^{2}}\mp \frac{iQ}{R_{0}}\right)\right]\left(e^{i\phi }\delta (\omega -\omega_{0})+e^{-i\phi }\delta (\omega +\omega_{0})\right), \end{array}$$

where cos(*ω*_0_*t* + *ϕ*) was expanded as an addition of exponentials and the definition for the Dirac delta function was used to solve the integral. The evaluation of ${\tilde{I}}_{\pm }$ goes into equation (2.6) to find

2.11 $$\tilde{S}(Q,\omega =\pm \omega_{0})=\frac{A}{2R_{0}}\left(\cos QR_{0}-\mathrm{sinc}\;QR_{0}\right),$$

where $\tilde{S}(Q,\omega )$ is zero for all other non-zero *ω*. The above equation is the classical model for $\tilde{S}(Q,\omega )$ from a diatomic undergoing simple harmonic motion.

Equation (2.11) has a notable feature. The amplitude of motion, *A*, does not contribute a spatial beat in $\tilde{S}(Q,\omega )$ for *A*≪1/*Q*_max_, and the prefactor *A*/2*R*_0_ is indistinguishable from the overall excitation fraction of this state. This suggests that *Q*_max_≥1/*A* is required to observe the amplitude of motion, and that experiments in the limit of low *Q*, i.e. *Q*_max_≪1/*A*, do not observe the amplitude of motion. To confirm the classical model, the discussion now returns to the experimental data.

For a Raman excitation, an amplitude of motion on the order of 0.2 Å is anticipated within the harmonic region of the potential. This would require *Q*_max_≥5 Å^−1^ to observe according to the classical model, so if the experiment is insensitive to the amplitude of motion, it should match the classical model. The classical model is compared to the measurement in figure 7. Between 1.81 and 4 Å^−1^, the model and measurement agree to within the experimental error. The statistical experimental error is calculated by finding the mean X-ray intensity, *I*(*x*, *y*, *τ*), at each pixel and across each pump-probe shot for that time-delay, and the variance of *I*(*x*, *y*, *τ*). This is then propagated through our analysis as described in appendix C. Below 1.81 Å^−1^, the signal is attenuated due to a physical beam block as shown in figure 3, and above 4 Å^−1^, the signal is not statistically significant due to few photon counts. This comparison supports the validity of the classical model and the existence of a limit on the ability to observe motion in a TRXS experiment analogous to the static limit of *d*≥2*π*/*Q*_max_. The comparison does not, however, inform us as to the *Q*_max_ required to observe the amplitude of motion.

To investigate the required *Q*_max_, the quantum description for the nuclear probability density is now considered. Consider the nuclear probability density generated by the overlap of the two low lying vibrational states of iodine, *ψ*_*v*_ and *ψ*_*v*+1_. These produce a charge density given by

2.12 $$\begin{array}{rl} \rho (R,\tau ) & ={\left|a\psi_{v}(R)\;e^{-iE_{v}\tau /\hbar }+b\psi_{v+1}(R)\;e^{-iE_{v+1}\tau /\hbar -i\phi }\right|}^{2}\\ & =a^{2}{\left|\psi_{v}(R)\right|}^{2}+b^{2}{\left|\psi_{v+1}(R)\right|}^{2}+2ab\cos(\omega_{0}\tau +\phi )\psi_{v}\psi_{v+1}, \end{array}$$

where *a*^2^ and *b*^2^ are the excitation fractions, *E*_*v*_ and *E*_*v*+1_ are the eigenenergies, *ω*_0_ = (*E*_*v*+1_ − *E*_*v*_)/ℏ, and *ϕ* is the relative phase of the states. Plugging equation (2.12) into equations (2.1) and (2.3), we find that

2.13 $$\tilde{S}(Q,\omega =\omega_{0})=\int dRR^{2}\;\mathrm{sinc}(QR)\psi_{v}(R)\psi_{v+1}(R).$$

This shows that TRXS spectrum is simply the momentum-space transform of *ψ*_*v*_(*R*)*ψ*_*v*+1_(*R*). (The coherent and incoherent contribution of different choices of *v* and *v* + 1 states, e.g. (*v* = 1, *v* + 1 = 2) versus (*v* = 2, *v* + 1 = 3), does not change the observed scattering at *Q* < 5 Å^−1^ as only *R*_0_ impacts the scattering at low *Q*. Above this threshold, the interference of these terms is evident.)

**Figure 8.**
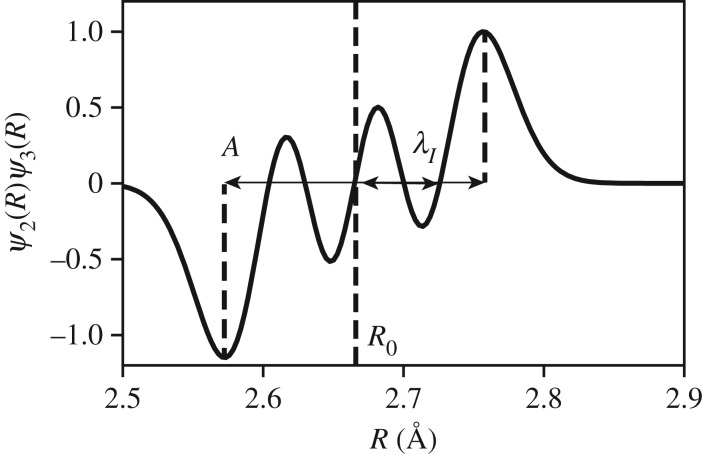
We show *ψ*_*v*_(*R*)*ψ*_*v*+1_(*R*) using *v* = 2 and *v* = 3. There are three notable spatial features of this wave function: the central position *R*_0_, the amplitude of oscillation *A* and the wavelength between nodes, *λ*_*I*_. Only *R*_0_ impacts the structure of scattering at low *Q* as shown in figure 9. Note that (*v* =  2, *v* + 1 = 3) are chosen for discussion not because they may be fingerprinted in the experimental data but because *ψ*_2_(*R*)*ψ*_3_(*R*) clearly highlights the difference between *A* and *λ*_*I*_.

**Figure 9.**
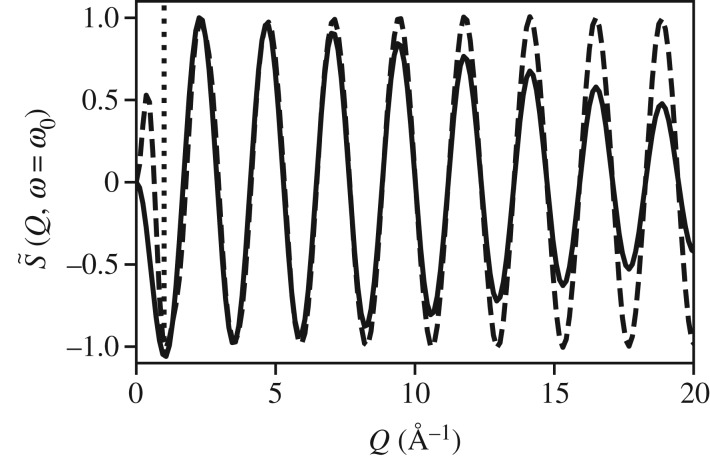
Comparison of the classical theory for $\tilde{S}(Q,\omega =\omega_{0})$ (dashed line) and the quantum mechanical model for the same system (solid line). We observe that the classical model has an artefact below 1 Å^−1^ due to the Dirac delta representation. Otherwise the two models agree up to about 7 Å^−1^ at which point we begin to observe changes that can only be attributed to the amplitude of motion and fine structure of the wavepacket as shown in figure 10.

**Figure 10.**
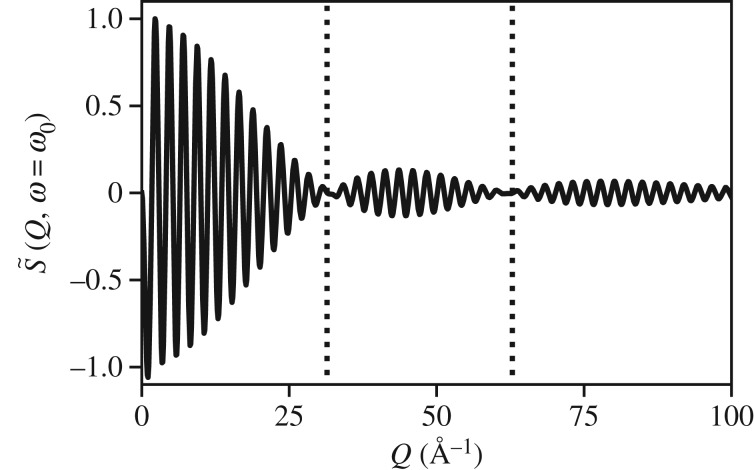
Extended range of $\tilde{S}(Q,\omega =\omega_{0})$ for the wave function shown in figure 8. Nodes exist at 2*π*/*A* and 4*π*/*A*, where *A* ≈ 0.2 Å.

Depending on the range of *Q*, $\tilde{S}(Q,\omega =\omega_{0})$ may obtain information on the equilibrium position, the amplitude of oscillation, or the width of the distribution as shown in figure 8. We observe in figure 9 that the classical and the quantum results agree in the limit of *Q* < 5 Å^−1^, and we observe in figure 10 that the amplitude of motion is embedded in the high *Q* limit. In particular, we observe nodes at *Q* = 2*π*/*A* and *Q* = 4*π*/*A* for *A* ≈ 0.2 Å. Therefore, the analogy to the static limit, *d*≥2*π*/*Q*_max_, for resolving harmonic motion of amplitude, *A*, is *A*≥2*π*/*Q*_max_.

## 3. Discussion and conclusion

**Figure 11.**
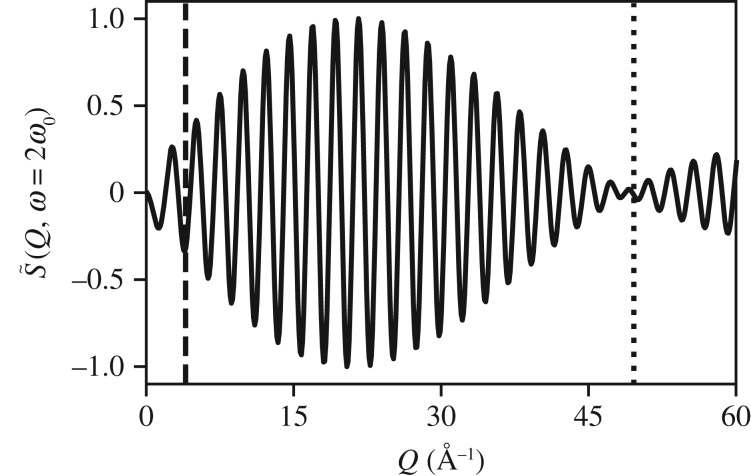
$\tilde{S}(Q,\omega =2\omega_{0})$ generated from *ψ*_1_(*R*)*ψ*_3_(*R*) and scaled relative to figure 10. Below 4 Å^−1^ (dashed line), the 2*ω*_0_ signal is no more than 25% of the scattering signal, but is clearly impacted by the amplitude of motion due to a *π* phase shift relative to the *ω*_0_ signal in figure 10. The node (dotted line) is at 3*π*/*A* for this state.

This does not rule out measuring an amplitude of *A* ≤ 2*π*/*Q*_max_, but to do so requires observing higher multiples of the fundamental frequency. The classical theory suggests these are small in the limit *Q*≪1/*A*. The quantum theory shows that scattering from *ψ*_*v*−1_(*R*)*ψ*_*v*+1_(*R*), i.e. the 2*ω*_0_ signal, is no stronger than 25% of the *ψ*_*v*_(*R*)*ψ*_*v*+1_(*R*) signal below 4 Å^−1^ as shown in figure 11, and that is before accounting for the relative excitation fractions of the states which would only further reduce the relative signal. That said, there is evident impact of the amplitude of motion on the scattering at low *Q* for states *ψ*_*v*−1_(*R*)*ψ*_*v*+1_(*R*), so with sufficient time-resolution and statistics even small oscillatory motions might be observable with TRXS.

This suggests that there are dual requirements on both *ω*_max_ and *Q*_max_ to observe the amplitude of oscillatory motion. For example, an experiment like the one described here with approximately 40 fs of temporal resolution measuring motion with a 155 fs period requires *Q*_max_ > 2*π*/*A* of resolution to measure the amplitude of motion. If instead the experiment had sufficient time resolution to measure the 2*ω*_0_ beat, the amplitude of motion might have been resolvable with the *Q*_max_ = 4.0 Å^−1^ of the experiment. It is in this second limit that our colleagues Biasin *et al.* [6] found themselves. While they had insufficient max *Q* to resolve the fine structure of the molecular state directly at the harmonic frequency, their time-resolution of ≈30 fs as compared to a period of 330 fs was such that the small amplitude of motion, 0.005 Å, may have been resolvable as shown.

In conclusion, the above considerations on *ω*_max_ and *Q*_max_ provide an analogy for the static imaging limit, *d*≥2*π*/*Q*_max_. For simple harmonic motion moving with frequency, *ω*_0_, and amplitude, *A*, about an equilibrium position, *R*_0_, *A*≥2*π*/*Q*_max_ is the smallest observable amplitude of motion for experiments with *ω*_max_ ≈ *ω*_0_. For experiments with *ω*_max_≥*ω*_0_, even smaller amplitudes of motion may be observable.

## Supplementary Material

phil-trans-data

## Data accessibility

Access to the full LINAC coherent light source (LCLS) datasets is available upon request. The reduced dataset projected onto Legendre polynomials, $S(Q,\tau )=\sum_{l}S_{l}(Q,\tau )P_{l}(\cos\theta )$, is included as electronic supplementary material. The analysis code is available at [17] with instructions for processing the full or reduced datasets above.

## Appendix A. Experimental detail

The experimental data were recorded at the LCLS at the X-Ray Pump Probe (XPP) end station [18] with an experimental setup that has already been described in detail elsewhere [4,5,7], and is shown diagrammatically in figure 2. An 800 nm optical laser with a pulse length of 70 fs, a bandwidth of 28 nm FWHM, and a focal intensity of >10^13^ W cm^−2^ was focused into a gas cell containing 100 °C iodine (50 torr, [19]). The gas cell had a holey mirror to collinearly combine the laser with 9 keV, 40 fs, 2 mJ, 30 μm FWHM X-rays from the LCLS. The forward scattered X-rays had a usable angular range of 23–58.2° [4] which corresponds to a *Q*-range of 1.81–4.43 Å^−1^ as shown in figure 3. The scattered photons were detected using the large area Cornell Stanford Pixel Area Detector (CSPAD) [18] located behind the cell. The XPP beamline's optical/X-ray cross correlator was used to get timing jitter below 15 fs root-mean-squared [20]. The optical pump delay was varied with respect to the X-rays using an encoded continuously moving translation stage moving delay back and forth between −0.5 ps and 2 ps delay to help average over slow fluctuations in experimental parameters such as beam pointing, position, X-ray intensity, etc.

The high intensity 800 nm laser was likely able to multi-photon ionize some of the iodine, and near *t*_0_ in the observed TRXS shown in figure 5, there is indication of the subsequent rapid dissociation in excess of 30 Å ps^−1^, see [7,21] for representative images of strong dissociation in TRXS). The estimate for the dissociation velocity arises from examination of the sloped line in figure 6, where the velocity should go as *v* = Δ*ω*/Δ*Q*. Some of the iodine were observed to undergo Raman transitions to higher lying vibrational states. The periodic motion in the observed X-ray scattering pattern is centred about the mean ground state iodine separation of *R*_0_ = 2.666 Å and oscillates with a period of 155.9 ± 3.9 fs as demonstrated in figures 6 and 7. These observations are consistent with previous experiments which have measured an equilibrium separation of 2.666 Å and a harmonic period of 155.5 fs [11–13].

## Appendix B. General theory of time-resolved X-ray scattering from homonuclear diatomics

In the limit of impulsive scattering, the time-dependent X-ray scattering intensity may be expressed as

B 1 $$\frac{dI}{d\Omega }=\frac{d\sigma_{Th}}{d\Omega }\int dkI(k)\frac{\omega }{\omega_{s}}\langle \psi (\tau )|\hat{F}(Q)|\psi (\tau )\rangle ,$$

where d*σ*_*Th*_/d*Ω* is the Thomson scattering cross-section, *I*(***k***) is the incident X-ray flux, |*ψ*(*τ*)〈 is the state of the molecule at pump-probe delay *τ* = *t* − *t*_0_, ***k*** is the incident X-ray momentum, ***k***_*s*_ is the scattered X-ray momentum, ***Q*** = ***k*** − ***k***_*s*_ is the momentum transfer and $\hat{F}(Q)=\sum_{j,l}e^{iQ\cdot (r_{j}-r_{l})}$ is the scattering operator, where ***r***_*j*,*l*_ are the electronic coordinates [22].

For scattering within the pulse bandwidth (*ω* ≈ *ω*_*s*_) and collimated beam input *I*_0_, equation (B 1) reduces to the product of three factors:

B 2 $$\frac{dI}{d\Omega }=\frac{d\sigma_{Th}}{d\Omega }I_{0}\langle F(Q,\tau )\rangle ,$$

where 〈*F*(***Q***, *τ*)〉 is a time- and angle-dependent polarization-corrected scattering probability. 〈*F*(***Q***, *τ*)〉 may subsequently be expressed as an incoherent sum of the scattering from each electronic state because the coherent cross-terms between electronic states in X-ray scattering are smaller than the incoherent contributions by several orders of magnitude (e.g. [23]). The incoherent contribution 〈*F*_*N*_(***Q***, *τ*)〉 from each electronic state *N* is simplified considerably by the independent atom approximation so that the scattering probability for a homonuclear diatomic molecule becomes a simple function of the atomic separation ***R*** [24]:

B 3 $$\langle F_{N}(Q,\tau )\rangle =2|f_{A}(Q){|}^{2}\left(1+Re\left\{\int dR\rho_{N}(R,\tau )\;e^{iQ\cdot R}\right\}\right).$$

Here *f*_*A*_(*Q*) is the atomic scattering factor and *ρ*_*N*_(***R***, *τ*) is the ensemble-averaged nuclear probability density of electronic state *N* at pump-probe delay *τ*. The exponential function in equation (B 3) can be rewritten using the spherical Bessel expansion:

B 4 $$e^{iQ\cdot R}=\sum_{l}i^{l}(2l+1)P_{l}(\cos\theta )j_{l}(QR),$$

where *j*_*l*_(*QR*) is the *l*th spherical Bessel function. The X-ray diffraction pattern projects onto Legendre polynomials that describe the target probability density [9,25]. For inversion-symmetric systems, e.g. the nuclear probability density following a dipole excitation, only *even* Legendre polynomials contribute:

B 5 $$S_{N}(Q,\theta ,\tau )=\sum_{l\;=\;0,2,\ldots }\sqrt{\frac{2\pi }{2l+1}}P_{l}(\cos\theta )S_{N,l}(Q,\tau ).$$

Only the isotropic contribution is considered in this manuscript as given by

B 6 $$F_{N,0}(Q,\tau )=2{\left|f_{A}(Q)\right|}^{2}\left(1+\int dRR^{2}\;\mathrm{sinc}(QR)\rho_{N}(R,\tau )\right).$$

In particular, the molecular scattering factor is discussed,

B 7 $$S_{N,0}(Q,\tau )=\frac{F_{N,0}}{2{\left|f_{A}(Q)\right|}^{2}}-1=\int dRR^{2}\;\mathrm{sinc}(QR)\rho_{N}(R,\tau ).$$

The subscripts have been dropped in the manuscript such that

B 8 $$S(Q,\tau )=\int dRR^{2}\;\mathrm{sinc}(QR)\rho (R,\tau )$$

is written for brevity.

To analyse the experimental data, these theoretical considerations are applied. The measured time-dependent X-ray scattering intensity on the CSPAD, *I*(*x*, *y*, *τ*), is used to generate the molecular scattering factor up to a scaling factor and DC offset via

B 9 $$F(x,y,\tau )=\frac{I(x,y,\tau )}{\left(d\sigma_{Th}/d\Omega )(x,y\right)}$$

and

B 10 $$S(x,y,\tau )=\frac{F(x,y,\tau )}{2{\left|f_{I}(x,y)\right|}^{2}}.$$

The isotropic component of *S*(*x*, *y*, *τ*) as given by *S*(*Q*, *τ*) is obtained by angularly integrating the data at fixed *Q* = 2*k*_0_sin*γ*/2 with respect to the angle, *θ*, about the laser polarization axis, ${\hat{e}}_{y}$. (These angles are depicted in figure 3.) The angular integration is done by projecting the data onto the zeroth-order Legendre function, i.e. *P*_0_(cos*θ*) = 1.

## Appendix C. Error propagation

For each shot, the pump-probe delay, *τ*′_*i*_, is measured and then binned into some time bin, *τ*_*j*_ ± Δ*τ*. This allows the generation of the mean molecular scattering pattern

C 1 $$S(x,y,\tau_{j})=\frac{1}{N_{j}}\sum_{i}S(x,y,\tau_{i}^{\prime }),$$

where (*x*, *y*) indicates each pixel on the CSPAD detector. The variation at each pixel position is generated by

C 2 $$\sigma^{2}(x,y,\tau_{j})=\mathrm{Var}\left(S(x,y,\tau_{j})\right)=\frac{1}{N_{j}}\sum_{i}{\left|S(x,y,\tau_{i}^{\prime })-S(x,y,\tau_{j})\right|}^{2}.$$

The variation is then propagated through the analysis as follows.

For both the isotropic projection of *S*(*x*, *y*, *τ*) onto *S*(*Q*, *τ*) and the temporal Fourier transform of *S*(*Q*, *τ*) onto $\tilde{S}(Q,\omega )$, a *χ*^2^-minimization is used. To perform the transforms

C 3 $$\chi^{2}=\sum_{l}\frac{{\left(p_{i}-s_{i}\right)}^{2}}{\sigma_{i}^{2}},$$

is minimized, where *p*_*i*_ is the fitted function, *s*_*i*_ is the data, and *σ*^2^_*i*_ is the variance. For a linear model, the fitted function may be expressed as $p_{i}=\sum_{j}x_{j}f_{j}(Q_{i})$, where *x*_*j*_ are the model coefficients. Then the solution to the *χ*^2^-minimization is

C 4 $$x_{k}=\sum_{i}\frac{A_{ki}s_{i}}{\sigma_{i}^{2}},$$

where *A* = (*f*^*T*^*σ*^−2^*f*)^−1^*f*. The associated error for the solution, *x*_*k*_, is then

C 5 $$\sigma_{k}\leq \sum_{j}\left|A_{kj}\sigma_{j}^{-1}\right|,$$

as shown in [26].

For the isotropic projection of *S*(*x*, *y*, *τ*) onto *S*(*Q*, *τ*), the fitted function is *f*_*j*_(*θ*_*i*_) = *P*_*j*_(cos*θ*_*i*_), where *j* = 0, 2, 4 are used to find a reduced *χ*^2^ of about 1. The obtained result is *x*_*j*_(*Q*, *τ*) = *S*_*j*_(*Q*, *τ*), where *j* = 0 is the desired isotropic contribution, denoted $\tilde{S}(Q,\omega )$ in the main text.

For the temporal Fourier transform of *S*(*Q*, *τ*) into $\tilde{S}(Q,\omega )$, the fitted function is *f*_*j*_(*τ*_*i*_) = cos(*ω*_*j*_*τ*_*i*_), where *ω*_*j*_∈(0, *π*/Δ*τ*) and Δ*τ* = 30 fs was used in this analysis.
